# (1*E*,4*E*)-1,5-Bis[2-(trifluoro­meth­yl)phen­yl]penta-1,4-dien-3-one

**DOI:** 10.1107/S1600536812051586

**Published:** 2013-01-04

**Authors:** Dong Ho Park, V. Ramkumar, P. Parthiban

**Affiliations:** a Department of Biomedicinal Chemistry, Inje University, Gimhae, Gyeongnam 621 749, Republic of Korea; b Department of Chemistry, IIT Madras, Chennai 600 036, TamilNadu, India

## Abstract

In the title compound, C_19_H_12_F_6_O, a monoketone derivative of curcumin, both double bonds have a *trans* conformation. The mol­ecule is mostly planar with all C and O atoms essentially coplanar, with the exception of one benzene ring, which is tilted by 17.18 (1)° with respect to the plane of the remainder of the mol­ecule. The r.m.s. deviation from planarity of the coplanar section is 0.0097 Å. The crystal packing features weak C—H⋯O and C—H⋯F inter­actions.

## Related literature

For the synthesis of chalcones, see: Tully *et al.* (2001 ▶). For the biological properties of chalcones, see: Buescher & Yang (2000 ▶); Kumar *et al.* (2003 ▶), Hsu & Cheng (2007 ▶). For their physical properties, see: Fichou *et al.* (1988 ▶); Butcher *et al.* (2006 ▶). For similar structures, see: Butcher *et al.* (2007 ▶); Nizam Mohideen *et al.* (2007 ▶); Harrison *et al.* (2006 ▶).
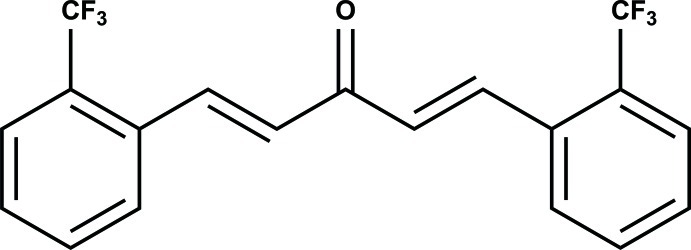


## Experimental

### 

#### Crystal data

C_19_H_12_F_6_O
*M* *_r_* = 370.29
Monoclinic, 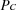

*a* = 11.3123 (12) Å
*b* = 4.7907 (4) Å
*c* = 15.1697 (16) Å
β = 101.834 (3)°
*V* = 804.63 (14) Å^3^
*Z* = 2
Mo *K*α radiation
μ = 0.14 mm^−1^
*T* = 298 K
0.35 × 0.25 × 0.10 mm

#### Data collection

Bruker APEXII CCD area-detector diffractometer
Absorption correction: multi-scan (*SADABS*; Bruker, 2004 ▶) *T*_min_ = 0.952, *T*_max_ = 0.986
5185 measured reflections
2608 independent reflections
1931 reflections with *I* > 2σ(*I*)
*R*_int_ = 0.020

#### Refinement

*R*[*F* ^2^ > 2σ(*F* ^2^)] = 0.037
*wR*(*F* ^2^) = 0.096
*S* = 1.04
2608 reflections
235 parameters
2 restraints
H-atom parameters constrained
Δρ_max_ = 0.20 e Å^−3^
Δρ_min_ = −0.14 e Å^−3^

### 

Data collection: *APEX2* (Bruker, 2004 ▶); cell refinement: *SAINT* (Bruker, 2004 ▶); data reduction: *SAINT*; program(s) used to solve structure: *SHELXS97* (Sheldrick, 2008 ▶); program(s) used to refine structure: *SHELXL97* (Sheldrick, 2008 ▶); molecular graphics: *ORTEP-3* (Farrugia, 2012) ▶; software used to prepare material for publication: *SHELXL97*.

## Supplementary Material

Crystal structure: contains datablock(s) global, I. DOI: 10.1107/S1600536812051586/zl2522sup1.cif

Structure factors: contains datablock(s) I. DOI: 10.1107/S1600536812051586/zl2522Isup2.hkl

Supplementary material file. DOI: 10.1107/S1600536812051586/zl2522Isup3.cml

Additional supplementary materials:  crystallographic information; 3D view; checkCIF report

## Figures and Tables

**Table 1:** Hydrogen-bond geometry (Å, °)

*D*—H⋯*A*	*D*—H	H⋯*A*	*D*⋯*A*	*D*—H⋯*A*
C17—H17⋯F3^i^	0.93	2.62	3.399 (4)	141 (2)
C1—H1⋯O1^ii^	0.93	2.72	3.290 (5)	121 (1)

Symmetry codes: (i) 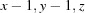; (ii) 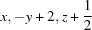.

**Fig. 1.**
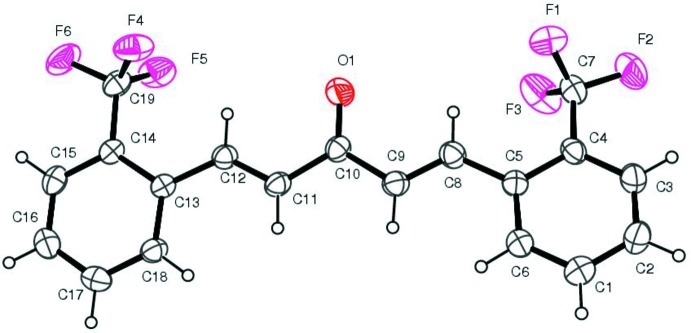
Anistropic displacement representation of the molecule with atoms represented with 30% probability ellipsoids.

**Fig. 2.**
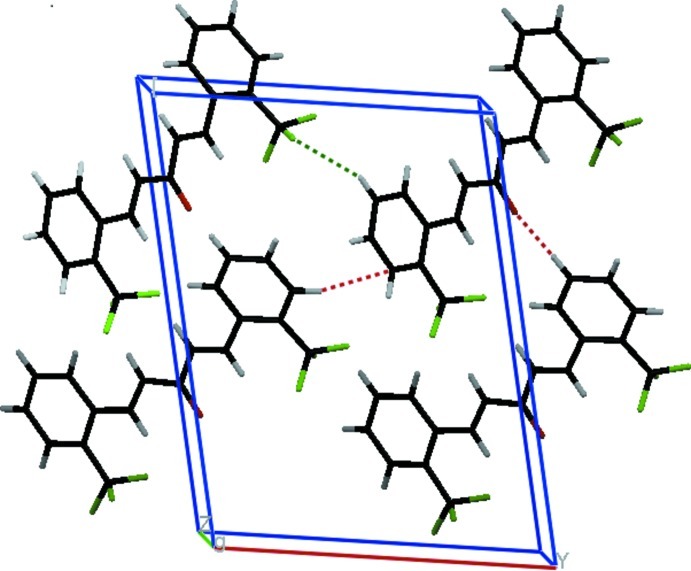
Packing diagram showing C—H···O, C—H···F and C—H···π interactions.

## supplementary crystallographic information

### Comment

The title compound is a bischalcone, and is a monoketone derivative of curcumin. Curcumin is a naturally abundant beta-diketone derived from the rizome of *curcuma longa* (Buescher & Yang, 2000). Numerous studies have shown that curcumin possesses multiple pharmacological properties. Several clinical trials of curcumin were carried out in patients with pancreatic cancer, multiple myeloma, rheumatoid arthritis, cystic fibrosis, inflammatory bowel disease, psoriasis, and other disorders (Kumar *et al.*, 2003; Hsu & Cheng, 2007). Since the stereochemistry of the synthesized molecule is an important criterion for its biological actions, it is of of desirable to establish the structure of the synthesized molecule.

Crystalline chalcone derivatives are also of interest due to their their second harmonic generation properties, particularly, their often are good blue light emitters. The NLO properties of the molecules are also associated with their molecular geometry (Fichou *et al.*, 1988; Butcher *et al.*, 2006), and accordingly, a single-crystal XRD study of the title bischalcone was undertaken to obtain detailed information on its molecular conformation.

In the title compound, C_19_H_12_F_6_O, both double bonds have *trans* configuration. The molecule is mostly planar with all carbon and oxygen atoms coplanar with the exception of one phenyl ring (C1—C6), which is tilted by 17.18 (1)° against the plane of the remainder of the molecule. The root mean square deviation from planarity of the coplanar section Ph—C=C—C(O)—C=C (C8—C18) is 0.0097 Å.

The bond lengths of the conjugated chalcone backbone C5—C13 [C5–C8 = 1.460 (4), C8–C9 = 1.322 (4), C9–C10 = 1.460 (4), C10–C11 = 1.470 (4), C11–C12 = 1.311 (4) and C12–C13 = 1.456 Å] show alternate localized single and double bonds as in its *ortho*-chloro analog (Nizam Mohideen *et al.*, 2007). This indicates the absence of delocalization of the double bonds in the chalcone skeleton C5—C13.

The crystal packing of this molecule is stabilized by weak intermolecular C—H···O, C—H···F and C—H···π interactions (Table 1).

### Experimental

The title compound, 1,5-bis(2-(trifluoromethyl)phenyl)penta-1,4-dien-3-one was synthesized by a modified procedure of Tully *et al.* (2001) using the milder base ammonium acetate instead of sodium hydroxide and a better yield (86%) was obtained. 20 mmol of 2-trifluoromethylbenzaldehyde (2.634 ml), 15 mmol of acetone (1.11 ml) and 1 g of ammonium acetate were combined in one-pot and stirred gently in ethanol as the solvent (20 ml). The complete consumption of the starting materials was monitored by TLC. After completion, the reaction mass was filtered, washed with cold ethanol and dried. The compound was characterized by melting point, IR and NMR. The analytical and spectral data are as reported previously (Tully *et al.*, 2001). X-ray diffraction quality crystals of the title compound were obtained by slow evaporation of an ethanol solution.

### Refinement

All hydrogen atoms were fixed geometrically and allowed to ride on the parent carbon atoms with C—H = 0.93 Å with *U*_iso_(H) = 1.2*U*_eq_(C).

### Crystal data

**Table:**

C_19_H_12_F_6_O	*F*(000) = 376
*M**_r_* = 370.29	*D*_x_ = 1.528 Mg m^−^^3^
Monoclinic, *P**c*	Mo *K*α radiation, λ = 0.71073 Å
Hall symbol: P -2yc	Cell parameters from 2360 reflections
*a* = 11.3123 (12) Å	θ = 2.7–24.1°
*b* = 4.7907 (4) Å	µ = 0.14 mm^−^^1^
*c* = 15.1697 (16) Å	*T* = 298 K
β = 101.834 (3)°	Block, colourless
*V* = 804.63 (14) Å^3^	0.35 × 0.25 × 0.10 mm
*Z* = 2	

### Data collection

**Table:**

Bruker APEXII CCD area-detector diffractometer	2608 independent reflections
Radiation source: fine-focus sealed tube	1931 reflections with *I* > 2σ(*I*)
Graphite monochromator	*R*_int_ = 0.020
phi and ω scans	θ_max_ = 27.8°, θ_min_ = 2.7°
Absorption correction: multi-scan (*SADABS*; Bruker, 2004)	*h* = −14→14
*T*_min_ = 0.952, *T*_max_ = 0.986	*k* = −5→5
5185 measured reflections	*l* = −18→18

### Refinement

**Table:**

Refinement on *F*^2^	Primary atom site location: structure-invariant direct methods
Least-squares matrix: full	Secondary atom site location: difference Fourier map
*R*[*F*^2^ > 2σ(*F*^2^)] = 0.037	Hydrogen site location: inferred from neighbouring sites
*wR*(*F*^2^) = 0.096	H-atom parameters constrained
*S* = 1.04	*w* = 1/[σ^2^(*F*_o_^2^) + (0.0351*P*)^2^ + 0.2469*P*] where *P* = (*F*_o_^2^ + 2*F*_c_^2^)/3
2608 reflections	(Δ/σ)_max_ < 0.001
235 parameters	Δρ_max_ = 0.20 e Å^−^^3^
2 restraints	Δρ_min_ = −0.14 e Å^−^^3^

### Special details

**Table:**

Geometry. All e.s.d.'s (except the e.s.d. in the dihedral angle between two l.s. planes) are estimated using the full covariance matrix. The cell e.s.d.'s are taken into account individually in the estimation of e.s.d.'s in distances, angles and torsion angles; correlations between e.s.d.'s in cell parameters are only used when they are defined by crystal symmetry. An approximate (isotropic) treatment of cell e.s.d.'s is used for estimating e.s.d.'s involving l.s. planes.
Refinement. Refinement of *F*^2^ against ALL reflections. The weighted *R*-factor *wR* andgoodness of fit *S* are based on *F*^2^, conventional *R*-factors *R* are basedon *F*, with *F* set to zero for negative *F*^2^. The threshold expression of*F*^2^ > σ(*F*^2^) is used only for calculating *R*-factors(gt) *etc*. and isnot relevant to the choice of reflections for refinement. *R*-factors basedon *F*^2^ are statistically about twice as large as those based on *F*, and *R*-factors based on ALL data will be even larger.

### Fractional atomic coordinates and isotropic or equivalent isotropic displacement parameters (Å^2^)

**Table:**

	*x*	*y*	*z*	*U*_iso_*/*U*_eq_	
C1	1.1775 (4)	1.5554 (9)	1.1292 (2)	0.0761 (11)	
H1	1.1342	1.5756	1.1747	0.091*	
C2	1.2832 (4)	1.6949 (8)	1.1343 (2)	0.0784 (11)	
H2	1.3110	1.8160	1.1819	0.094*	
C3	1.3481 (3)	1.6560 (7)	1.0689 (2)	0.0673 (9)	
H3	1.4220	1.7461	1.0736	0.081*	
C4	1.3062 (3)	1.4847 (6)	0.9956 (2)	0.0501 (7)	
C5	1.1957 (2)	1.3456 (6)	0.98898 (19)	0.0466 (7)	
C6	1.1349 (3)	1.3854 (7)	1.0575 (2)	0.0619 (9)	
H6	1.0619	1.2933	1.0549	0.074*	
C7	1.3802 (3)	1.4515 (8)	0.9268 (2)	0.0654 (10)	
C8	1.1460 (3)	1.1721 (7)	0.9110 (2)	0.0533 (8)	
H8	1.1829	1.1877	0.8618	0.064*	
C9	1.0547 (3)	0.9955 (7)	0.9024 (2)	0.0508 (8)	
H9	1.0168	0.9688	0.9507	0.061*	
C10	1.0119 (3)	0.8412 (7)	0.8190 (2)	0.0492 (7)	
C11	0.9120 (3)	0.6426 (6)	0.81579 (19)	0.0505 (8)	
H11	0.8802	0.6177	0.8671	0.061*	
C12	0.8662 (3)	0.4996 (6)	0.7433 (2)	0.0478 (7)	
H12	0.9004	0.5317	0.6934	0.057*	
C13	0.7685 (2)	0.2965 (6)	0.73090 (18)	0.0420 (6)	
C14	0.7286 (2)	0.1566 (6)	0.64946 (17)	0.0428 (7)	
C15	0.6361 (3)	−0.0368 (6)	0.6405 (2)	0.0540 (8)	
H15	0.6105	−0.1284	0.5859	0.065*	
C16	0.5820 (3)	−0.0946 (7)	0.7110 (2)	0.0617 (9)	
H16	0.5208	−0.2272	0.7047	0.074*	
C17	0.6184 (3)	0.0439 (7)	0.7910 (2)	0.0595 (8)	
H17	0.5806	0.0084	0.8388	0.071*	
C18	0.7107 (3)	0.2348 (7)	0.80077 (19)	0.0518 (8)	
H18	0.7351	0.3253	0.8557	0.062*	
C19	0.7851 (3)	0.2061 (7)	0.5705 (2)	0.0578 (8)	
F1	1.3299 (2)	1.5590 (5)	0.84801 (15)	0.0944 (7)	
F2	1.4881 (2)	1.5699 (6)	0.94957 (17)	0.1057 (9)	
F3	1.4031 (2)	1.1853 (6)	0.9114 (2)	0.1095 (9)	
F4	0.7781 (2)	0.4718 (5)	0.54360 (14)	0.0860 (7)	
F5	0.89964 (19)	0.1390 (5)	0.58521 (14)	0.0902 (7)	
F6	0.7316 (2)	0.0638 (5)	0.49807 (13)	0.0954 (7)	
O1	1.0559 (2)	0.8784 (5)	0.75344 (16)	0.0824 (8)	

### Atomic displacement parameters (Å^2^)

**Table:**

	*U*^11^	*U*^22^	*U*^33^	*U*^12^	*U*^13^	*U*^23^
C1	0.083 (3)	0.090 (3)	0.058 (2)	−0.016 (2)	0.0204 (19)	−0.011 (2)
C2	0.096 (3)	0.076 (3)	0.059 (2)	−0.025 (2)	0.005 (2)	−0.0118 (19)
C3	0.063 (2)	0.073 (2)	0.060 (2)	−0.0199 (18)	−0.0003 (17)	0.0054 (19)
C4	0.0462 (15)	0.0481 (18)	0.0545 (18)	−0.0042 (14)	0.0065 (13)	0.0089 (15)
C5	0.0479 (17)	0.0427 (17)	0.0481 (17)	0.0016 (13)	0.0076 (14)	0.0024 (14)
C6	0.061 (2)	0.068 (2)	0.058 (2)	−0.0144 (16)	0.0168 (16)	−0.0079 (17)
C7	0.057 (2)	0.067 (3)	0.073 (2)	−0.0142 (19)	0.0140 (17)	−0.0020 (19)
C8	0.0503 (17)	0.054 (2)	0.0572 (19)	−0.0016 (15)	0.0149 (14)	−0.0019 (15)
C9	0.0577 (18)	0.0486 (18)	0.0474 (17)	−0.0004 (14)	0.0138 (14)	−0.0002 (13)
C10	0.0544 (18)	0.0456 (19)	0.0466 (17)	0.0013 (14)	0.0080 (14)	−0.0025 (14)
C11	0.0614 (19)	0.0500 (19)	0.0408 (17)	−0.0002 (15)	0.0124 (14)	−0.0058 (13)
C12	0.0562 (18)	0.0440 (18)	0.0439 (16)	−0.0005 (14)	0.0119 (14)	−0.0004 (13)
C13	0.0470 (15)	0.0385 (16)	0.0404 (15)	0.0054 (13)	0.0089 (12)	−0.0002 (12)
C14	0.0504 (16)	0.0353 (16)	0.0416 (16)	0.0080 (13)	0.0070 (13)	−0.0015 (12)
C15	0.0589 (18)	0.0463 (18)	0.0511 (18)	0.0045 (16)	−0.0021 (15)	−0.0066 (15)
C16	0.0523 (18)	0.056 (2)	0.074 (2)	−0.0040 (16)	0.0067 (16)	0.0041 (17)
C17	0.0603 (19)	0.060 (2)	0.062 (2)	0.0020 (17)	0.0219 (16)	0.0124 (17)
C18	0.0622 (19)	0.0524 (19)	0.0420 (17)	−0.0007 (15)	0.0134 (14)	−0.0028 (14)
C19	0.070 (2)	0.056 (2)	0.0466 (19)	0.0075 (17)	0.0097 (16)	−0.0062 (16)
F1	0.1087 (16)	0.114 (2)	0.0642 (14)	−0.0084 (15)	0.0272 (12)	0.0071 (13)
F2	0.0714 (14)	0.133 (2)	0.120 (2)	−0.0401 (15)	0.0359 (13)	−0.0138 (16)
F3	0.1025 (17)	0.0725 (17)	0.176 (3)	0.0065 (13)	0.0802 (17)	−0.0137 (16)
F4	0.135 (2)	0.0631 (15)	0.0670 (12)	0.0083 (14)	0.0377 (12)	0.0116 (11)
F5	0.0785 (14)	0.1231 (19)	0.0772 (13)	0.0270 (13)	0.0352 (11)	−0.0012 (14)
F6	0.1393 (18)	0.0984 (17)	0.0467 (11)	−0.0143 (15)	0.0144 (12)	−0.0244 (12)
O1	0.0925 (18)	0.0963 (19)	0.0667 (16)	−0.0401 (16)	0.0356 (14)	−0.0247 (14)

### Geometric parameters (Å, º)

**Table:**

C1—C2	1.358 (5)	C10—C11	1.470 (4)
C1—C6	1.366 (5)	C11—C12	1.310 (4)
C1—H1	0.9300	C11—H11	0.9300
C2—C3	1.362 (5)	C12—C13	1.456 (4)
C2—H2	0.9300	C12—H12	0.9300
C3—C4	1.385 (4)	C13—C18	1.386 (4)
C3—H3	0.9300	C13—C14	1.397 (4)
C4—C5	1.401 (4)	C14—C15	1.383 (4)
C4—C7	1.474 (5)	C14—C19	1.488 (4)
C5—C6	1.372 (4)	C15—C16	1.364 (4)
C5—C8	1.460 (4)	C15—H15	0.9300
C6—H6	0.9300	C16—C17	1.371 (5)
C7—F1	1.318 (4)	C16—H16	0.9300
C7—F2	1.326 (4)	C17—C18	1.373 (4)
C7—F3	1.331 (4)	C17—H17	0.9300
C8—C9	1.321 (4)	C18—H18	0.9300
C8—H8	0.9300	C19—F5	1.309 (4)
C9—C10	1.460 (4)	C19—F6	1.329 (4)
C9—H9	0.9300	C19—F4	1.334 (4)
C10—O1	1.213 (4)		
			
C2—C1—C6	120.1 (4)	C9—C10—C11	118.2 (3)
C2—C1—H1	119.9	C12—C11—C10	122.4 (3)
C6—C1—H1	119.9	C12—C11—H11	118.8
C1—C2—C3	119.4 (3)	C10—C11—H11	118.8
C1—C2—H2	120.3	C11—C12—C13	128.1 (3)
C3—C2—H2	120.3	C11—C12—H12	115.9
C2—C3—C4	121.2 (3)	C13—C12—H12	115.9
C2—C3—H3	119.4	C18—C13—C14	117.2 (3)
C4—C3—H3	119.4	C18—C13—C12	120.6 (2)
C3—C4—C5	119.5 (3)	C14—C13—C12	122.3 (3)
C3—C4—C7	118.8 (3)	C15—C14—C13	120.6 (3)
C5—C4—C7	121.7 (3)	C15—C14—C19	118.0 (3)
C6—C5—C4	117.3 (3)	C13—C14—C19	121.4 (3)
C6—C5—C8	121.5 (3)	C16—C15—C14	120.8 (3)
C4—C5—C8	121.2 (3)	C16—C15—H15	119.6
C1—C6—C5	122.4 (3)	C14—C15—H15	119.6
C1—C6—H6	118.8	C15—C16—C17	119.6 (3)
C5—C6—H6	118.8	C15—C16—H16	120.2
F1—C7—F2	105.8 (3)	C17—C16—H16	120.2
F1—C7—F3	106.2 (3)	C16—C17—C18	120.1 (3)
F2—C7—F3	104.7 (3)	C16—C17—H17	120.0
F1—C7—C4	113.4 (3)	C18—C17—H17	120.0
F2—C7—C4	113.3 (3)	C17—C18—C13	121.8 (3)
F3—C7—C4	112.7 (3)	C17—C18—H18	119.1
C9—C8—C5	127.5 (3)	C13—C18—H18	119.1
C9—C8—H8	116.3	F5—C19—F6	106.4 (3)
C5—C8—H8	116.3	F5—C19—F4	106.3 (3)
C8—C9—C10	121.6 (3)	F6—C19—F4	104.4 (3)
C8—C9—H9	119.2	F5—C19—C14	113.4 (3)
C10—C9—H9	119.2	F6—C19—C14	112.7 (3)
O1—C10—C9	121.2 (3)	F4—C19—C14	112.9 (3)
O1—C10—C11	120.7 (3)		
			
C6—C1—C2—C3	−2.3 (6)	O1—C10—C11—C12	−0.3 (5)
C1—C2—C3—C4	2.4 (6)	C9—C10—C11—C12	178.6 (3)
C2—C3—C4—C5	−0.9 (5)	C10—C11—C12—C13	179.6 (3)
C2—C3—C4—C7	179.7 (4)	C11—C12—C13—C18	0.5 (4)
C3—C4—C5—C6	−0.7 (4)	C11—C12—C13—C14	−179.6 (3)
C7—C4—C5—C6	178.6 (3)	C18—C13—C14—C15	−0.7 (4)
C3—C4—C5—C8	177.5 (3)	C12—C13—C14—C15	179.5 (3)
C7—C4—C5—C8	−3.1 (4)	C18—C13—C14—C19	−179.5 (3)
C2—C1—C6—C5	0.7 (6)	C12—C13—C14—C19	0.6 (4)
C4—C5—C6—C1	0.8 (5)	C13—C14—C15—C16	0.0 (4)
C8—C5—C6—C1	−177.4 (3)	C19—C14—C15—C16	178.9 (3)
C3—C4—C7—F1	−113.3 (3)	C14—C15—C16—C17	1.1 (5)
C5—C4—C7—F1	67.3 (4)	C15—C16—C17—C18	−1.5 (5)
C3—C4—C7—F2	7.4 (5)	C16—C17—C18—C13	0.8 (5)
C5—C4—C7—F2	−172.0 (3)	C14—C13—C18—C17	0.2 (4)
C3—C4—C7—F3	126.0 (3)	C12—C13—C18—C17	−179.9 (3)
C5—C4—C7—F3	−53.4 (4)	C15—C14—C19—F5	−117.0 (3)
C6—C5—C8—C9	−13.8 (5)	C13—C14—C19—F5	61.9 (4)
C4—C5—C8—C9	168.1 (3)	C15—C14—C19—F6	4.0 (4)
C5—C8—C9—C10	178.0 (3)	C13—C14—C19—F6	−177.1 (3)
C8—C9—C10—O1	−2.9 (5)	C15—C14—C19—F4	121.9 (3)
C8—C9—C10—C11	178.3 (3)	C13—C14—C19—F4	−59.2 (4)

### Hydrogen-bond geometry (Å, º)

**Table:**

*D*—H···*A*	*D*—H	H···*A*	*D*···*A*	*D*—H···*A*
C17—H17···F3^i^	0.93	2.62	3.399 (4)	141 (2)
C1—H1···O1^ii^	0.93	2.72	3.290 (5)	121 (1)
C3—H3···*Cg*1	0.93	3.32 (2)	4.096 (3)	142 (1)

Symmetry codes: (i) *x*−1, *y*−1, *z*; (ii) *x*, −*y*+2, *z*+1/2.
